# The Applicability and Limitations of the Spectrofluorometric Method for Determination of ALDH1 Activity in Serum and Plasma

**DOI:** 10.3390/diagnostics14232721

**Published:** 2024-12-03

**Authors:** Sylwia Michorowska, Agnieszka Wiśniewska, Renata Wolinowska, Piotr Wroczyński, Joanna Giebułtowicz

**Affiliations:** 1 Department of Drug Chemistry, Pharmaceutical and Biomedical Analysis, Medical University of Warsaw, Banacha 1, 02-097 Warsaw, Poland; sylwia.solobodowska@wum.edu.pl (S.M.); piotr.wroczynski@wum.edu.pl (P.W.); 2 Department of Laboratory Medicine, Medical University of Warsaw, Banacha 1, 02-097 Warsaw, Poland; agnieszka.wisniewska@wum.edu.pl; 3 Department of Pharmaceutical Microbiology and Bioanalysis, Medical University of Warsaw, Banacha 1, 02-097 Warsaw, Poland; renata.wolinowska@wum.edu.pl

**Keywords:** plasma, serum, ALDH, aldehyde dehydrogenase, fluorometric method

## Abstract

Background: Aldehyde dehydrogenase class 1 (ALDH1) is an enzyme that is ubiquitously distributed in adult tissues and may serve as a prognostic marker in various cancer types. In blood, 99% of ALDH1 is found in erythrocytes; although, it was also demonstrated that leukocytes and platelets exhibit ALDH activity. No ALDH activity was detected in plasma, even when employing the highly sensitive fluorometric method with 7-methoxy-1-naphthaldehyde as a substrate. However, some reports have been released describing stable and measurable ALDH1 activity in the serum of healthy subjects using 6-methoxy-2-naphthaldehyde as a substrate and a Shimadzu RF—5301 spectrofluorometer. Methods: Our study aimed to verify whether ALDH1 activity can be measured in plasma or serum (*n* = 80) using 6-methoxy-2-naphthaldehyde as a substrate and a highly sensitive Hitachi F7000 spectrofluorometer, which offers a higher signal-to-noise ratio compared to the Shimadzu RF-5301. Additionally, HPLC with fluorometric detection was used to validate the results (*n* = 25) and analyze the influence of hemolysis (*n* = 5) and liver cell damage (*n* = 15) on ALDH1 activity in serum. Results: Measurable ALDH activity in serum/plasma was very rarely detected using a spectrofluorometer (2 cases out of 80). However, background drift in assays without coenzyme addition was observed, and it may be easily mistaken for ALDH or oxidase activity. Therefore, the spectrofluorometer drift observed in blank assays and modified by a matrix, e.g., enhanced in protein-rich samples, should be considered in ALDH1 activity assays. Conclusions: The spectrofluorometric method has limited applicability for determining ALDH activity in plasma and serum. HPLC can measure ALDH1 activity in plasma or serum; however, factors like hemolysis and elevated liver enzymes significantly affect activity and must be considered in diagnostic interpretations. To enhance research quality on ALDH1 as a biomarker for diseases, including cancers, we recommend using control samples, reference materials, and purifying commercially available aldehyde substrates to improve method sensitivity.

## 1. Introduction

Aldehyde dehydrogenase (ALDH) is a polymorphic enzyme responsible for the oxidation of various endogenous and exogenous aldehydes to their corresponding carboxylic acids, utilizing NAD^+^ and/or NADP^+^ as cofactors [1].

In recent decades, research has consistently shown that abnormal expression or activity of ALDHs is implicated in various human diseases, both tumorous [1] and non-tumorous [2]. Examples of non-tumorous conditions include Alzheimer’s disease [3], cardiovascular diseases [4], alcohol intolerance [5], and certain pregnancy complications such as intrauterine growth restriction [6]. ALDH-related disorders can also result from genetic variations in the *ALDH* genes, particularly single-nucleotide polymorphisms. Examples include Sjögren–Larsson syndrome [7], keratoconus [8], diabetes mellitus in hypertensive patients [9], hemorrhagic stroke [10], arteriosclerosis in multiple arteries [11], and hyperprolinemia type II [12]. Aberrant ALDH activity or expression has been identified in a range of cancers, including breast cancer [13], lung cancer [14], head-and-neck squamous-cell carcinoma [15], prostate cancer [16], bladder cancer [17], and glioblastoma [18]. Accumulating evidence highlights ALDH1 as a significant cancer stem cell biomarker associated with tumor development, metastasis, and poor prognosis across various cancer types, including breast cancer [19], prostate cancer [20], cervical cancer [21,22,23], liver [24], colorectal cancer [25], and ovarian cancer [26,27].

The enzymatic activities of ALDH1, which include the production of retinoic acid and the detoxification of reactive byproducts, are crucial for stem cell functions for two main reasons. Firstly, even stem cells in hypoxic environments rely partially on oxidative metabolism, producing reactive oxygen species. Secondly, retinoic acid receptors play a key role in tissue development, such as embryogenesis and regeneration, particularly in morphogenesis and clonogenicity [28,29,30,31]. Consequently, ALDH1A1 targeting can be important in an anticancer treatment, e.g., in acute myeloid leukemia [32], ovarian cancer [33], and triple-negative breast cancer [34].

ALDH1 is ubiquitously distributed in the adult epithelium of the testis, brain, eye lens, kidney, and lungs. It is also found in the liver, pancreas, and stomach mucosa. In blood, 99% of ALDH1 is located in erythrocytes, but it was also demonstrated that leukocytes and platelets possess ALDH activity [35]. No ALDH activity was detected in plasma by Helander and Tottmar [35]. Similarly, Wierzchowski, using a highly sensitive fluorometric method with 7-methoxy-1-naphthaldehyde as a substrate, found neither ALDH nor oxidase activity in normal human plasma (at a 100-fold effective dilution) [36]. Moreover, the analysis of blood samples of patients suffering from various diseases (*n* = 30) revealed measurable ALDH activity in serum only in three cases (after liver transplantation, in acute circulatory arrest, and in acute renal failure) [37].

Recently, some reports have been released describing stable and measurable ALDH1 activity in the serum of healthy subjects, ranging from 1.48 to 7.06 mU/L, using 6-methoxy-2-naphthaldehyde as a substrate and a Shimadzu RF—5301 spectrofluorometer [38,39,40,41]. Therefore, the aim of our studies was to verify whether ALDH1 activity is measurable in plasma/serum (*n* = 80) using a highly sensitive spectrofluorometer Hitachi F7000, which has a higher signal-to-noise ratio compared to the Shimadzu RF—5301, with 6-methoxy-2-naphthaldehyde as a substrate. To validate the results, we also employed a more sensitive high-performance liquid chromatography (HPLC) method (*n* = 25) and analyzed the influence of hemolysis (*n* = 5) and liver cell damage (*n* = 15) on ALDH1 activity in serum. Moreover, we obtained recombinant ALDH1A1 protein, and we used it to confirm our hypothesis. 

## 2. Materials and Methods

### 2.1. Samples Collection

Serum and plasma EDTA samples from a cohort of 80 patients were analyzed using the spectrofluorometric method. Among these, one sample with undetectable ALDH1 activity was also subjected to HPLC analysis. HPLC was further used to measure ALDH activity in additional samples categorized as follows: (1) without elevated liver enzymes (LDH, AspT, and AlAT) and without visible signs of hemolysis (*n* = 25); (2) without elevated liver enzymes (LDH, AspT, and AlAT) but with visible signs of hemolysis (*n* = 5); and (3) with elevated liver enzymes (LDH, AspT, and AlAT) and without visible signs of hemolysis (*n* = 15). A drawback of assessing ALDH1 activity in body fluids is that the enzyme loses its activity after freezing (our unpublished results showed that when aldehyde dehydrogenases are present outside cells, such as in saliva, plasma, or serum, standard freezing at −80 °C leads to a complete loss of activity in the sample). These samples were obtained from residual blood following routine diagnostic procedures at the Public Central Teaching Hospital in Warsaw. All specimens were de-identified and stored in unmarked test tubes at refrigerated conditions. ALDH activity was assessed on the day of sample collection.

### 2.2. Recombinant ALDH1A1

Recombinant ALDH1A1 was obtained using the full-length human *ALDH1A1* gene amplified from TrueClone cDNA clone in a pCMV6-AC vector purchased from OriGene. The sequences of the 5′ and 3′ PCR primers were as follows: 5′CTAGCTAGCATGTCATCCTCAGGCACG3′ and 5′CCGGAATTCTTATGAGTTCTTCTGAGAGAT3′, respectively. A NheI site was introduced by the PCR primer on the 5′ end, whereas a EcoRI site was introduced on the 3′ end. The resulting 1.5 kb PCR-amplified fragment was digested with NheI and EcoRI, gel-purified using the QIAquick Gel Extraction Kit (Qiagen, Hilden, Germany), and ligated with a pET-28a vector (Novagene, Beijing, China), which has been digested with the same restriction enzymes and gel-purified. The product of the ligation reaction was transformed into *E. coli* BL21 (DE3) competent cells (Invitrogen, Waltham, MA, USA). The sequence of the entire insert of the pEt-28a-ALDH1A1 plasmid was verified by sequencing. The cultures of the overproducing strain were grown at 37 °C in LB broth (35 g/L tryptone, 20 g/L yeast extract, and 5 g/L NaCl, Sigma-Aldrich, St. Louis, MO, USA), supplemented with 50 μg/mL kanamycin to an OD_600_ of 0.6. The expression was induced by adding IPTG (isopropyl β-D-1-thiogalactopyranoside, Sigma-Aldrich) to a final concentration of 1 mM. Recombinant ALDH1A1 was isolated with a Ni-NTA Fast Start Kit (Qiagen, Hilden, Germany) according to the manufacturer’s guidelines and dialyzed overnight to 50 mM pyrophosphate buffer containing 1 mM EDTA and 1 mM DDT (Sigma-Aldrich) to prevent the loss of enzyme activity.

### 2.3. Spectrofluorometric Method of ALDH1 Determination

ALDH1 activity was determined as described previously [42]. Briefly, fluorometric assays were performed in a 50 mM pyrophosphate buffer, pH 8.1, at 25 °C, in the presence of 1 mM EDTA and 0.5 mM DTT. The assays utilized a highly fluorogenic naphthaldehyde substrate, 6-methoxy-2-naphthaldehyde, MONAL (5 µM) (Sigma-Aldrich, St. Louis, MO, USA), reacting with NAD^+^ (100 µM) (Sigma-Aldrich, St. Louis, MO, USA) as a co-substrate. All assays were run in the presence of 5 mM 4-methylpyrazole, 4-MP, an alcohol dehydrogenase inhibitor. 6-Methoxy-2-naphthoic acid, MONCO (1.5 µM) (Sigma-Aldrich), was used as a standard. The assay was also performed without NAD^+^ addition to account for non-specific MONAL oxidation. The ALDH activity was calculated using the difference between the fluorescence increase with NAD^+^ (representing specific and non-specific oxidation) and without NAD^+^ addition (representing non-specific oxidation). Furthermore, recombinant ALDH1A1 activity was measured in lower NAD^+^ concentration (50 nM) in order to verify whether low coenzyme concentration (which can be intrinsically present in serum/plasma) can result in an increase in fluorescence in a blank fluorometric assay (serum/plasma without NAD^+^ addition). As it is suggested that NAD^+^ concentration in mammalian serum is around 100 nM [43], the amount of cofactor added in assays with recombinant protein was high enough to guarantee physiological levels in 15-fold and 600-fold diluted biological samples (6.67 nM and 0.17 nM, respectively) and even to simulate the possible release of NAD^+^ from erythrocytes due to minor and invisible hemolysis, which can occur during the preanalytical phase. Another reason for using a higher cofactor concentration is the fact that increased NAD^+^ concentrations in the extracellular fluids can occur under specific conditions (e.g., infection (660 nM)) and in selected cell types (fibroblast and epithelial cell lines have the ability to steadily release NAD^+^) [44,45].

The recombinant ALDH activity was also measured without NAD^+^ addition to check the level of background fluorescence and/or spectrofluorometer drift. The samples were examined at two dilutions, 15-fold and 600-fold. The increase in the fluorescence of the naphthoate was recorded at 360 nm, with excitation at 315 nm, for 10 min using a Hitachi F7000 spectrofluorometer. Additionally, changes in MONCO concentration (measured at the start and after 30 min of the reaction) were measured using HPLC on 15-fold diluted samples.

The spectrofluorometer is calibrated annually by a certified service provider. Additionally, before conducting any experiments, the response intensity of the fluorescent reaction product is tested.

### 2.4. Chromatographic Method of ALDH1 Determination

HPLC was performed using a Shimadzu chromatograph consisting of an LC-10AD pump and RF10AXL fluorescence detector. Twenty microliters of the deproteinized reaction mixtures were introduced onto the column. Separation was performed on the Supelcosil LC-18-DB 25 cm × 4.6 mm, 5 µm column (Supelco, Bellefonte, PA, USA), under isocratic conditions. The mobile phase consisted of an acetonitrile–water mixture (65:35, *v*/*v*) at a pH of 2.8. The chosen pH provided a well-shaped peak, while the composition of the mobile phase allowed for rapid analyte elution without any detectable interference. The mobile phase was pumped at a flow rate of 1 mL/min. Chromatography was carried out at 30 °C. Fluorescence excitation and emission wavelengths were set at 310 and 360 nm, respectively. All eluents were of HPLC purity grade.

### 2.5. Statistical Analysis

The results are expressed as means ± standard deviations. Statistical comparisons were performed using the paired Student’s *t*-test or ANOVA test (multiple comparisons). A significance level of *p* < 0.05 was considered statistically significant. Correlation analysis was conducted using Spearman’s correlation coefficient to assess relationships between variables. Statistical analyses were performed using Statistica 10 software (Statsoft, Krakow, Poland).

## 3. Results

**Figure 1:**
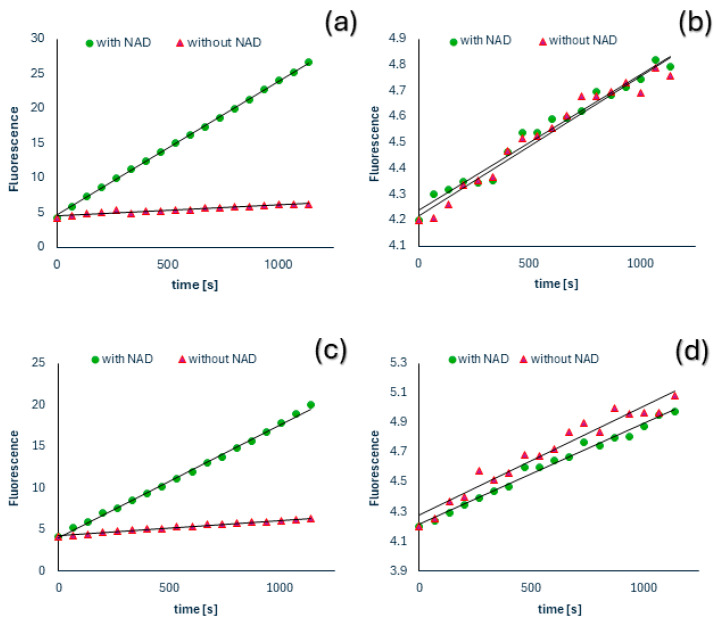
Kinetics of fluorescence increase in plasma (**a**,**b**) and serum (**c**,**d**) samples from two exemplar patients, with measurements taken both with (green circles) and without (red triangles) NAD^+^ addition. One of the two cases in which measurable ALDH activity was found in both plasma (**a**) and serum (**c**) is illustrated. The mean slopes and their standard deviations are as follows: (**a**) (19.2 ± 0.1) × 10^3^ FU/s with NAD^+^ and (1.7 ± 0.6) × 10^3^ FU/s without NAD^+^; (**b**) (0.47 ± 0.07) × 10^3^ FU/s with NAD^+^ and (0.49 ± 0.06) × 10^3^ FU/s without NAD^+^; (**c**) (13.7 ± 0.7) x10^3^ FU/s with NAD^+^ and (1.8 ± 0.1) × 10^3^ FU/s without NAD^+^; (**d**) (0.62 ± 0.09) × 10^3^ FU/s with NAD^+^ and (0.677 ± 0.003) × 10^3^ FU/s without NAD^+^.

Spectrofluorometric measurements revealed that, except for two patients, ALDH activity was generally undetectable in both serum and plasma samples. The calculated limit of detection and quantitation for the method are approximately 48 and 157 mU/L, respectively. The two patients exhibited ALDH activity levels of 5030 ± 1300 mU/L and 210 ± 30 mU/L in plasma and 3400 ± 170 mU/L and 170 ± 10 mU/L in serum. It is important to note that these values were adjusted to account for the baseline measurement obtained without NAD^+^ addition, indicating specific oxidation levels (Figure 1). The assay conducted without NAD^+^ addition showed an increase in fluorescence corresponding to an activity of approximately 450 ± 240 mU/L in plasma and 510 ± 310 mU/L in serum for the 15-fold dilution and 1500 ± 560 mU/L and 2480 ± 770 mU/L for the 600-fold dilution, respectively. The slopes were significantly higher in serum than in plasma (15-fold dilution *p* < 0.003; 600-fold dilution *p* < 0.00001). However, in the first case, the paired sample sign test revealed no statistically significant difference between these two matrices. A notable correlation was observed between the fluorescence increments in plasma and serum samples (15-fold dilution r = 0.69, *p* < 0.00001; 600-fold dilution r = 0.82, *p* < 0.00001). The two patients with measurable ALDH1 activity also showed increased liver enzymes activities. Another reason for the elevated ALDH1 activity could be sample hemolysis.

**Figure 2:**
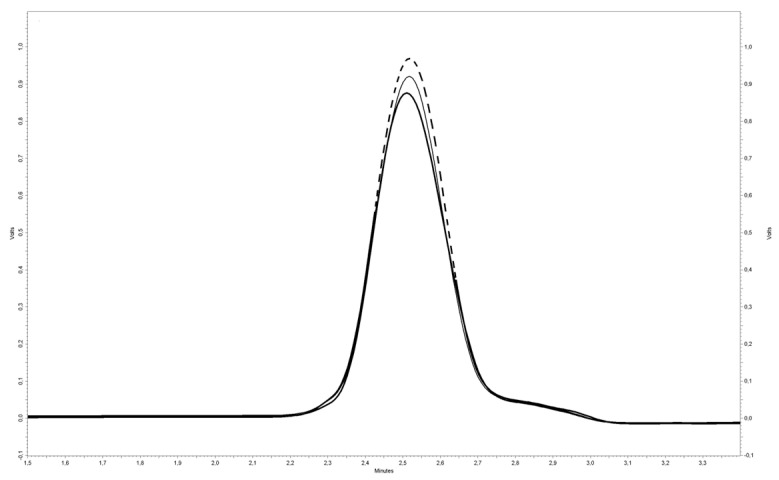
An exemplar chromatogram is presented for the serum sample, which demonstrated the highest measured ALDH activity, yet was still classified as having immeasurable activity by a spectrofluorometer. The peak shown corresponds to MONCO. The black line represents the sample at t = 0, the grey line represents the sample after 30 min of reaction without NAD^+^, and the dashed line represents the sample with NAD^+^ after 30 min of reaction. It is noteworthy that a prominent peak is observable even at time zero, likely attributed to the low oxidative stability of the aldehyde during storage. The marginal increase in peak area observed in the absence of NAD^+^ can be attributed to the low activity of other enzymes that do not require NAD^+^ as a cofactor, such as aldehyde oxidase.

Next, one of the samples already subjected to spectrofluorometric readings, which demonstrated the highest ALDH activity, yet was still classified as having immeasurable activity by the spectrofluorometer, was subjected to HPLC analysis. This sample showed the activity of 6.0 mU/L (Figure 2). Due to the inability to reanalyze all the samples previously tested spectrofluorometrically (because of the instability of ALDH1 and the lack of capability for long-term storage of biological material), a new group of patients was selected for HPLC analysis. In response to our concerns about the potential impact of hemolysis and/or liver diseases on the results, we investigated the ALDH1 activities in patient serum samples under the following conditions: (1) without elevated liver enzymes (LDH, AspT, and AlAT) and without visible signs of hemolysis; (2) without elevated liver enzymes (LDH, AspT, and AlAT) but with visible signs of hemolysis; and (3) with elevated liver enzymes (LDH, AspT, and AlAT) and without visible signs of hemolysis. HPLC analysis showed that ALDH1 activity in the first group was 5.5 ± 4.3 mU/L, with two samples having unmeasurable activities. Concerning non-specific activity (no NAD^+^ addition), almost 70% of serum samples had unmeasurable activities. The average activity in the remaining samples was 4.5 ± 0.8 mU/L. Hemolysis affected ALDH1 activity in serum, resulting in a mean of 101 ± 97 mU/L (*p* < 0.0001). Additionally, significantly higher ALDH1 activity was observed in the serum of patients with elevated liver enzymes, with a mean of 52 ± 36 mU/L (*p* < 0.0001).

**Figure 3:**
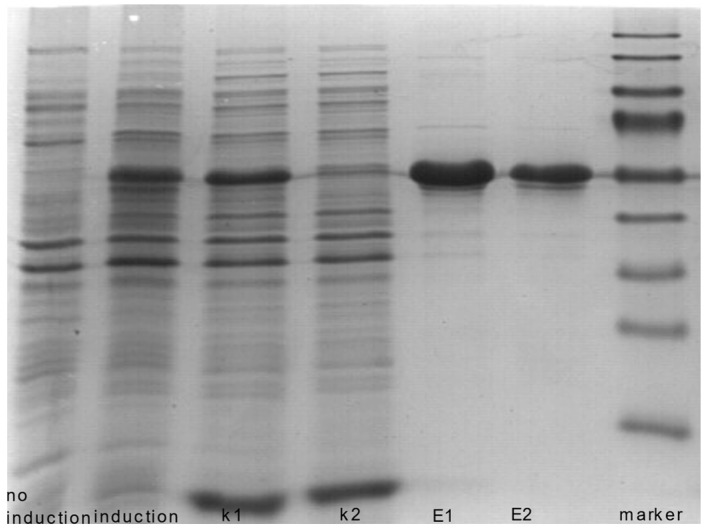
Expression and purification of the recombinant ALDH1A1 protein analyzed by SDS-PAGE (polyacrylamide gel electrophoresis) with Coomassie staining. Recombinant protein, with a molecular weight of 55 kD, was expressed as a fusion protein. Lane ‘marker’: pre-stained molecular weight markers (PageRuler, Fermentas, Vilnius, Lithuania) with the following masses: 17, 26, 34, 43, 55, 72, 95, 130, and 170 kDa; lane ‘no induction’: extracts from bacteria containing pET-28a-ALDH1A1 before IPTG induction; lane ‘induction’: extracts from bacteria containing pET-28a-ALDH1A1 after IPTG induction; lane ‘k1’: pellet from centrifugation of bacterial cell lysate after IPTG induction, before purification with His-binding columns; lane ‘k2’: flowthrough from the bacteria extract during purification with the His-binding columns; lanes E1 and E2: purified recombinant protein following purification with His-binding columns.

We confirmed the expression of recombinant ALDH1A1 in *E. coli* by SDS-PAGE. The SDS-PAGE analysis of recombinant ALDH1A1 showed a protein band of the expected size in recombinant bacteria (Figure 3). His-tagged ALDH1A1 was successfully purified using His-binding columns (Figure 3). The predicted molecular weight of ALDH1A1 is 55 kDa, and the molecular weight of the His tag is 3.5 kDa. We confirmed the identity of the fusion protein using Western blotting with rabbit monoclonal antibodies (Abcam, Cambridge, UK). The results indicated that ALDH1A1 was successfully expressed. However, to maintain enzymatic activity, the protein had to be dialyzed and immediately frozen in liquid nitrogen.

**Figure 4:**
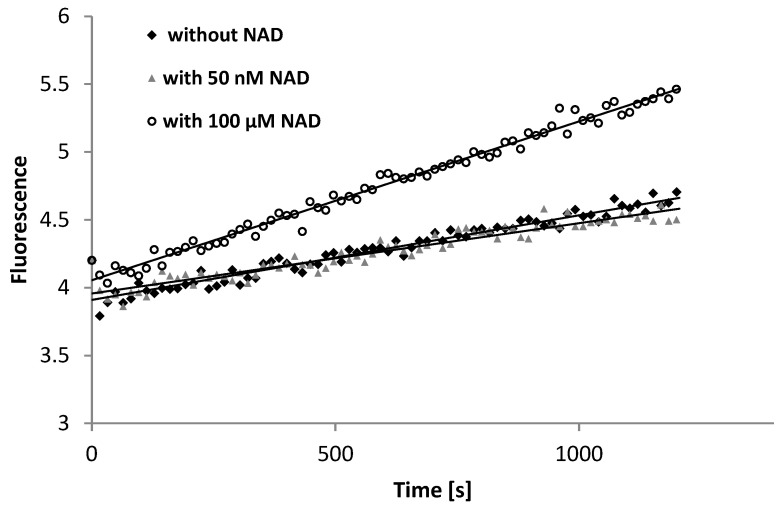
The course of fluorescence increase obtained for the recombinant protein, with an activity of 50 mU/L. The mean slopes (and their standard deviations) are as follows: (0.60 ± 0.10) × 10^3^ FU/s without NAD^+^, (0.47 ± 0.03) × 10^3^ FU/s with 50 nM NAD^+^, and (1.1 ± 0.2) × 10^3^ FU/s with 100 μM NAD^+^.

The recombinant ALDH1A1 protein was added to the pure buffer to eliminate potential interferences from the serum/plasma matrix while ensuring optimal conditions for the sensitive enzymatic assay. Analysis of the activity of recombinant ALDH1A1 revealed an increase in fluorescence even in the absence of NAD^+^. The increase was comparable to that observed at low concentration of NAD^+^ (50 nM), which can be detected in plasma (Figure 4). Thus, we concluded that the increase in fluorescence observed in blank serum/plasma samples may not be attributed to the activity of ALDH1A1 due to NAD^+^ intrinsically present in serum or plasma samples.

The differences were observed between the buffer containing 50 nM NAD^+^ and the buffer containing 100 µM NAD^+^ (typically used in the enzymatic assays) at such low enzyme activity levels as 50 ± 11 mU/L (Figure 4). Nevertheless, this activity was still approximately 10-times higher than that determined by the HPLC method. Thus, we diluted the enzyme solution 10 times. At the activity level of about 5.0 ± 3.0 mU/L (similar to the average ALDH activity level found in healthy patients using the HPLC method), the increase in fluorescence was comparable to that observed in the blank assay (*p* > 0.05) and was definitely below the quantification limit. We also checked whether the level of MONCO corresponding to the enzyme activity of 1.5–30 mU/L can be distinguished from a blank assay. ANOVA showed no statistically significant differences between the signal generated by the blank (increase due to water addition (0.15 ± 0.02 FU)) and the signals generated by the mentioned MONCO concentrations (increase of 0.07–0.16 FU).

## 4. Discussion

The most popular assays for measuring aldehyde dehydrogenase activity in clinical material are based on direct spectrophotometric determination due to the simplicity and widespread availability of spectrophotometers [46]. However, such analyses have two major disadvantages: significant background drift and low sensitivity. Alternatively, the HPLC method can be used for separation of the reaction products, but it is relatively expensive and time-consuming [47]. There are two fluorometric procedures available for detecting class I ALDH activity. The first procedure employs 7-methoxy-1-naphtaldehyde, which is oxidized to highly fluorescent 7-metoxy-1-naphthoate at a rate similar to that of acetaldehyde oxidation. Alternatively, another fluorogenic naphthaldehyde substrate, 6-methoxy-2-naphthaldehyde, may be used to assess the ALDH1 activity. However, this method is less recommended due to substrate’s tendency to form highly fluorescent complexes with plasma proteins, leading to increased background fluorescence. Additionally, 6-methoxy-2-naphthaldehyde may be a substrate for alcohol dehydrogenase, necessitating the addition of 4-MP to the reaction mixture [2]. It is worth noting that only 6-methoxy-2-naphthaldehyde is commercially available.

The increase in fluorescence observed in biological samples without NAD^+^ addition (using a spectrofluorometer) may result from one of the following: (1) aldehyde oxidase or other oxidoreductase activity (observed in HPLC assay), (2) ALDH1 activity at low NAD^+^ concentrations that are physiologically present in plasma/serum, (3) spectrofluorometer drift enhanced by the samples’ matrix, or 4) nonenzymatic oxidation of MONAL. Experiments with recombinant protein have excluded the first two possibilities as the only causes of the increase in fluorescence in biological samples without NAD^+^ addition. This conclusion is supported by the observation that even in a sample containing only buffer with recombinant ALDH1A1, an increase in fluorescence was noted. Moreover, as expected, a low NAD^+^ concentration of 50 nM did not result in detectable ALDH activity. We do not consider the fluorescence increase observed in spectrofluorometric determination of ALHD1 activity to result from non-enzymatic oxidation of MONAL (the fourth possibility), as the reaction mixture contained a potent antioxidant, DTT. HPLC analysis confirmed that potential non-specific oxidation of the enzyme substrate is indeed observed very rarely, and at such a low level that it would be undetectable by the spectrofluorometer. ALDH activity in serum of healthy people was very low, sometimes undetectable, even when using a highly concentrated sample (effective dilution of 15-fold), long reaction time of 30 min, and a highly sensitive technique such as HPLC. The observed low ALDH1 activity using HPLC in healthy group may also be attributed to undetectable hemolysis, given that ALDH1 is present in erythrocytes. Indeed, hemolysis and elevated liver enzymes are factors that strongly influence ALDH1 activity in serum. Nevertheless, as demonstrated in our study using recombinant protein, low enzyme activities detected in HPLC analyses of samples from healthy groups are undetectable by the spectrofluorometric method when using commercially available MONAL. It cannot be ruled out that purifying MONAL may increase the sensitivity of the method. The range of ALDH1 activity detected by the HPLC method, as well as the observed spectrofluorimetric drift, corresponds to concentrations reported in the literature [38]. However, here we showed that these low activities (below 50 mU/L) cannot be detected by the spectrofluorimetric method. As our team co-developed the described spectrofluorimetric method, we acknowledge responsibility for previously published materials that may not sufficiently highlight the risk of misinterpreting spectrofluorometric drift as ALDH activity in plasma and serum. Therefore, we have decided to address this gap.

The significant increases in fluorescence observed without NAD^+^ addition in patients’ plasma and serum may be a result of spectrofluorometer drift affected by matrix content/composition, e.g., protein concentration. The problem of spectrofluorometer drift and changes in sensitivity due to aging of the lamps, photomultipliers, and electronic components have been discussed in previous papers [48]. Therefore, it is essential to implement not only standards, but also reference materials for operational qualification and performance validation of these instruments. Given the potential for producing enzymatically active ALDH1A1 through genetic recombination methods, establishing control samples to measure ALDH1 activity would be justified to ensure that the obtained results are comparable across laboratories. This standardization is especially crucial as ALDH1 may serve as an important marker for various cancers.

Our results suggest that measurable ALDH activity is rarely observed using the spectrofluorometric method in serum/plasma. Moreover, spectrofluorometer drift, which was observed in blank assays, is exacerbated in protein-rich samples and could be mistaken for ALDH1 activity. HPLC is more suitable for measuring such low levels of activity. Factors such as hemolysis and elevated liver enzymes significantly influence ALDH1 activity in serum and should be considered when interpreting the results. To improve the quality of research regarding the use of ALDH1 as a biomarker for various diseases, including cancers, we proposed to incorporate control samples and reference material in measurements, as well as purifying commercially available aldehyde substrate to increase the sensitivity of the method.

## Data Availability

Data available on request.
